# Oropharyngeal cancer patient stratification using random forest based-learning over high-dimensional radiomic features

**DOI:** 10.1038/s41598-021-92072-8

**Published:** 2021-07-07

**Authors:** Harsh Patel, David M. Vock, G. Elisabeta Marai, Clifton D. Fuller, Abdallah S. R. Mohamed, Guadalupe Canahuate

**Affiliations:** 1 Department of Electrical and Computer Engineering, University of Iowa, Iowa City, 52242 USA; 2 Division of Biostatistics, University of Minnesota, Minneapolis, 55455 USA; 3 Department of Department of Computer Science, University of Illinois at Chicago, Chicago, 60607 USA; 4 Department of Radiation Oncology, MD Anderson Cancer Center, Houston, 77030 USA

**Keywords:** Cancer, Computational biology and bioinformatics

## Abstract

To improve risk prediction for oropharyngeal cancer (OPC) patients using cluster analysis on the radiomic features extracted from pre-treatment Computed Tomography (CT) scans. 553 OPC Patients randomly split into training (80%) and validation (20%), were classified into 2 or 3 risk groups by applying hierarchical clustering over the co-occurrence matrix obtained from a random survival forest (RSF) trained over 301 radiomic features. The cluster label was included together with other clinical data to train an ensemble model using five predictive models (Cox, random forest, RSF, logistic regression, and logistic-elastic net). Ensemble performance was evaluated over the independent test set for both recurrence free survival (RFS) and overall survival (OS). The Kaplan–Meier curves for OS stratified by cluster label show significant differences for both training and testing (*p* val < 0.0001). When compared to the models trained using clinical data only, the inclusion of the cluster label improves AUC test performance from .62 to .79 and from .66 to .80 for OS and RFS, respectively. The extraction of a single feature, namely a cluster label, to represent the high-dimensional radiomic feature space reduces the dimensionality and sparsity of the data. Moreover, inclusion of the cluster label improves model performance compared to clinical data only and offers comparable performance to the models including raw radiomic features.

## Introduction

Radiomics entails extraction of quantitative imaging features from computed tomography (CT), magnetic resonance imaging (MRI), or positron emission tomography (PET) images. A large number of radiomic features can be extracted from these images to characterize tumor intensity, shape, and texture. Dimensionality reduction can significantly reduce the number of features which represent the high-dimensional radiomic space. Dimensionality reduction seeks to identify tumor signature profiles that can be used for prognostic or predictive evaluation of patient outcomes^1,2^, and have been putatively associated with clinical and survival outcomes^3–6^.

Dimensionality reduction can be applied to select a subset of existing features or to generate a new feature space that summarizes the original high-dimensional feature space. Dimensionality reduction has been successfully used on a number of studies dealing with radiomic data^7–9^. Feature clustering can be used to reduce radiomics dimensionality^7,10,11^. Clustering can be used to represent an entire set of radiomic features and massively reduce the radiomic feature space into a single covariate^12^. The cluster label also allows easy visualization and differentiation of the patients^13^,^14^,^15^, which is difficult with feature selection alone.

In supervised dimensionality reduction, the outcome of interest is considered when producing a radiomic signature. Some studies have examined the use of unsupervised methods for event prediction with radiomic data^16,17^, but the inclusion of an outcome in the dimension reduction process has the potential to increase predictive power.

Survival endpoints, such as overall survival (OS), local recurrence control (LC), distant metastasis (DM), regional recurrence control (RC), or combined outcomes such as recurrence free survival (RFS) are considered right-censored when the time-to-event is unknown at the end of an individual’s follow-up. That is, at any given point during follow-up, some patients are yet without an event but still potentially at risk for an event with further follow-up. Samples for which the outcome has not been observed at the last follow up are said to be right-censored. Several standard machine learning applications have been extended to allow the use of right-censored data^18^. Some methods (e.g., random survival forests) have been developed to perform feature selection using the right-censored outcomes directly; that is, these methods directly account for the unequal follow-up time among individuals^8^.

### Objective

This paper focuses on developing a novel methodology for leveraging clustering over a high-dimensional set of radiomic features using random survival forest. The cluster label is used in posterior analyses to represent the entire radiomic feature space. Random forest (RF) is an increasingly popular approach for dealing with high dimensional data. A random forest is an ensemble-based decision tree method used for classification and feature selection. Random forests have been adapted to extend beyond a categorical outcome; random survival forests (RSF)^19^ use the right-censored outcome directly. Specifically, we propose using the proportion of times a pair of patients fall into the same terminal nodes in the trees of the random forest as a similarity metric to cluster the patients. This method is known as random forest clustering^20^, but previous studies^21,22,23^ have used random forest clustering for unsupervised learning to cluster unlabeled data. Our work differs from this previous work in that we are applying this to already labeled survival data to extract a single covariate, which can then be used to build predictive models. We use selected features and a trained regression model to assign previously unseen test samples into a cluster. Subsequently, the cluster label is used as a covariate for risk prediction from an ensemble model of established risk prediction approaches (Cox Proportional Hazard, Random Forest, Random Survival Forest, Logistic Regression, and Logistic-Elastic Net), which have been adapted to right-censored outcomes using inverse probability of censoring weights^18^.

## Materials and methods

### Data source

Our institutional database was retrospectively reviewed for oropharyngeal cancer patients treated at MD Anderson Cancer Center during the period of (2005–2013) following Institutional Review Board (IRB) approval. Eligible patients diagnosed with oropharyngeal cancers were pathologically confirmed either by a biopsy or a surgical excision and received their treatment (i.e., chemo-radiotherapy) with curative intent.

For imaging data, contrast-enhanced computed tomography (CECT) at initial diagnosis -before any active local or systemic treatment- were exported to our commercially available contouring software (Velocity AI v3.0.1). The volumes of interest (VOIs), including the gross primary tumor volumes (GTVp), were manually segmented by a radiation oncologist in a 3D fashion, then inspected by a second radiation oncologist. The generated VOIs and CT images were exported in the format of DICOM and DICOM-RTSTRUCT to be used for radiomics features extraction. The primary tumor volumes (GTVp) were contoured based on the ICRU 62/83 definition^24^. All methods were carried out in accordance with relevant guidelines and regulations. This retrospective study was approved by IRB, and in compliance with the Health Insurance Portability and Accountability Act (HIPAA), informed consent was waived and approved by the IRB as all analyses were performed over retrospective data.

### Radiomics analysis

Radiomics analysis was performed by the use of the freely available open-source software “Imaging Biomarker Explorer” (IBEX), which was developed by the University of Texas MD Anderson Cancer Center and utilized the MATLAB platform (MathWorks Inc, Natick, VA). The CT images in the format of DICOM and the GTVp contours in the format DICOMRTSTRUCT were imported into IBEX. We extracted features that represent intensity, shape, and texture of a tumor. The categorization of these features was ranked as first, second, and higher texture features based on the applied method from pixel to pixel^23^. More than 3800 radiomic features were considered in this analysis.

From these radiomic features, we removed those with zero variance and those with a correlation above 99% using the training dataset. Previous studies have identified tumor volume and intensity as relevant features for local control and other clinical outcomes^3,25,26,27^. To further reduce redundancy, we also removed any radiomic features that were highly correlated (> 80%) to the features: F25.ShapeVolume and F29.IntensityDirectGlobalMean. Ultimately these resulted in a remaining 301 radiomic features that were used for the proximity computation^3^.

### Clinical data preprocessing

As clinical data we consider age (continuous), HPV status (Positive/Negative/Unknown), Smoking status (Current/Former/Never), T-category 2 groups (T1-2/T3-4), N-category 2 groups (N0-1/N2-3), Therapeutic Combination (CC, IC + CC, IC + Radiation Alone, Radiation Alone), and AJCC staging (8th edition).

**Figure 1:**
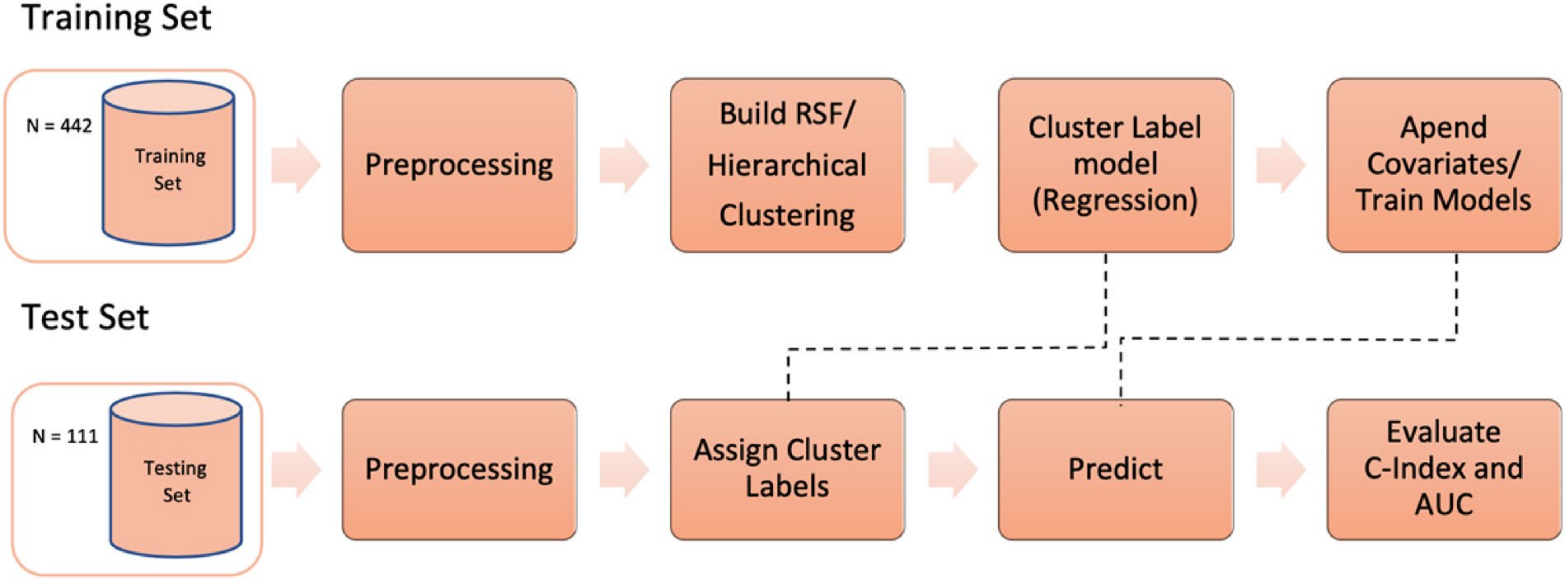
Processing pipeline overview. The data is split into disjoint training and validation (test) sets. Initially the data is preprocessed (remove radiomics with zero variance and highly correlated features, normalization, and clinical data categorization) and then the patients are clustered using Random Survival Forest (RSF) clustering. A regression model is trained using the cluster label as dependent variable and later used to assign test patients into a cluster. The ensemble model is trained using clinical covariates and the cluster labels and evaluated over the test data using the discrimination metrics C-Index and AUC.

*Methodological Development.* Figure 1 shows the overall processing pipeline, including the procedures for dimensionality reduction, evaluation, and cluster explanations. 80% of the sample was used in the training set, and 20% of samples in the test set.

### Random forest clustering

Using the training samples, we fit a random survival forest with the radiomic features as the possible predictors and the right-censored time-to-recurrence as the outcome, i.e., overall or recurrence free survival. We computed the proximity matrix from the random survival forest’s fit, i.e., the proportion of times two subjects fall into the same terminal node. Proximities computed for the training set are based on in-bag proximity, i.e., only considering the patients selected across all bootstrap samples. We decided to use in-bag instead of the default out-of-bag samples, because during clustering we are not using the random survival forests as a predictive model but rather to compute the similarity between two very high-dimensional samples.

The proximity matrix can be considered a similarity matrix and converted into a dissimilarity measure by subtracting it from the unit matrix. This dissimilarity matrix is then used for clustering, and the clustering algorithm that we use must consider only distances between points and not their absolute positions. Hierarchical clustering^28^ is a greedy approach where clusters are built either by starting with one large cluster and splitting it apart (divisive) or starting with a cluster for each point and then merging them at each step (agglomerative). We used the agglomerative approach along with the proximity matrix in our approach. With the matrix, we take the two most similar subjects and cluster them together. Distance between clusters may be measured several ways, and in this study, we used ward^29^, which is calculated with the following equation:

1 $$\delta \left( {c_{1} ,c_{2} } \right) = \frac{{\left| {c_{1} } \right|\left| {c_{2} } \right|}}{{\left| {c_{1} } \right| + \left| {c_{2} } \right|}}~\left| {\left| {c_{1} - c_{2} } \right|} \right|^{2}$$

$\delta$ is variance where the goal is to optimize it by minimizing the change, or the error sum of squares. The final extracted feature is simply the resulting cluster label from hierarchical clustering. Survival curves for subjects in each cluster were estimated using the Kaplan–Meier estimator.

### Cluster assignment for validation patients

After clustering, validation is done using a holdout test set, where test patients are not part of the original clustering. To assign a cluster label to the test samples, we train a regression model over the most important variables from the RSF using a Multinomial Log-Linear Model (mulitnom) over the training dataset to predict the cluster label for the validation patients. Multinomial regression was used instead of the classic binary logistic regression because we want to allow testing for more than 2 clusters.

### Leveraging cluster labels into survival prediction

To assess the added value of the radiomics clusters to predicting survival outcomes beyond standard clinical and demographic characteristics, we compare the performance of a predictive model using only clinical covariates with the same model including both clinical covariates and the cluster label. We fit an ensemble model using various regression and machine-learning-based models (Cox Proportional Hazard, Random Survival Forest, Random Forest, Logistic Regression, and Logistic-Elastic Net). The first two models are able to handle right-censored outcomes directly, while the later three require a binary outcome. We consider 5-year survival as the event outcome. Only patients that experienced the event before the 5-year cutoff are considered as positive samples. These models have been adapted to right-censored outcomes using inverse probability of censoring weights^30^ and patients without sufficient follow-up time that have not experienced the event have zero weight.

#### Ensemble model for survival prediction

These prediction models were combined into an ensemble model using stacking. We generated a stacked regression model using the base models' predictions as features and minimizing the prediction error. We use fivefold cross-validation over the training set to learn the values for the individual models’ coefficients (weights) to create the ensemble model. Using the individual model predictions from when each sample was in the test fold, we learn the coefficients that would minimize the square error of the prediction using the non-negative least squares (NNLS) method based on the Lawson–Hanson algorithm and the dual method of Goldfarb from the Superlearner R package^31^.

The performance of the ensemble model was assessed using the hold-out test set. In addition to the model using clinical data only, we compare performance to the models including clinical data and AJCC Staging, and a set of raw radiomic features selected using two different supervised methods: Random Survival Forest^32^ and Coxnet^33^. For the Random Survival Forest, we use the top features ranked by variable importance (highest frequencies). We use 1000 trees and a default node size of 2. To account for the randomness of the survival forest, we averaged the results after running ten times. The other feature selection method is a Cox Proportional Hazards Model using Regularization Paths for Generalized Linear Models (glm) via Coordinate Descent (coxnet)^33^. We use cross-validation over the training dataset to find the optimal value for the regularization coefficient and then use it to train the coxnet over the entire dataset and select the features with non-zero coefficients from the model. We use the term COX to represent these features. Two metrics of discrimination are used to evaluate the predictions for all the models: the area under the receiver operating curve (AUC)^34^ to predict 5-year survival and Harrel’s C-index^35^.

## Results

Table 1 summarizes the clinical and demographic characteristics of the 533 patients who met the inclusion criteria for this study. The split of training (442) and testing (111) is shown. The cohort was predominately male (~ 87% for both sets) and the median age was 58 and 56 for training and testing, respectively. Over half of the cohort (> 60% for both sets) was HPV positive. ~ 20% of patients died during follow-up and ~ 18% experienced a relapse.

**Table 1:** Data demographics.

Name	Train (442)	Test (111)
**Covariates**		
*Gender*		
Male	388 (87.8%)	97 (87.4%)
Female	54 (12.2%)	14 (12.6%)
Age at diagnosis (years)	58.2 (52.5–65.8)	56.6 (52.5–65.8)
* T category*		
T1/T2	277 (62.7%)	69 (62.2%)
T3/T4	165 (37.3%)	42 (37.8%)
* N category*		
N0/N1	226 (51.1%)	59 (53.2%)
N2/N3	216 (48.8%)	52 (46.8%)
* AJCC stage (8th edition)*		
I	153 (34.6%)	42 (37.8%)
II	82 (18.6%)	17 (15.3%)
III	57 (12.9%)	9 (8.1%)
IV	150 (33.9%)	43 (38.8%)
* Smoking status*		
Former	158 (35.8%)	44 (39.7%)
Current	92 (20.8%)	26 (23.4%)
Never	192 (43.4%)	41 (36.9%)
*Therapeutic combination*		
CC	228 (51.6%)	68 (61.3%)
IC + CC	119 (26.9%)	26 (23.4%)
IC + radiation alone	44 (10.0%)	10 (9.0%)
Radiation alone	51 (11.5%)	7 (6.3%)
* HPV status*		
Positive	270 (61%)	64 (60%)
Negative	41 (9%)	9 (8%)
Unknown	131 (29%)	39 (35%)
**Response**		
* Vital status (at end of follow-up)*		
Alive	355 (80.3%)	89 (80.2%)
Deceased	87 (19.7%)	22 (19.8%)
Survival time in months	65.4 (45.9–98.7)	75.3 (48.3–98.1)
*Relapse free survival*		
Alive	363 (82.1%)	91 (82.0%)
Deceased	79 (17.9%)	20 (18.0%)
Survival time in months	61.0 (40.6–96.4)	69.4 (39.3–94.8)

The table shows the demographics for the clinical covariates used in this study. The dataset (533 patients) was randomly split into training and testing disjoint sets using a 80–20 split. As expected, the same distributions can be observed for the train (442 patients) and test (111 patients) datasets. Within the cells in the table, the reported number is either: count (frequency %) for categorical/discrete covariates, or median (25th–75th percentiles) for continuous covariates.

The Random Survival Forest (RSF) was built over the training data and log-rank was used as the splitting rule, with a minimum node size of 5 as previously used to predict Parkinson’s disease with radiomic data^36^. The number of trees per forest was set to 1000. The co-occurrence matrix was extracted from the RSF and hierarchical clustering was used to identify 2–4 groups. Overall, the clusters were more balanced for OS than for RFS. For 2 clusters, the split was roughly 50–50% for OS and 70–30% for RFS.

**Figure 2:**
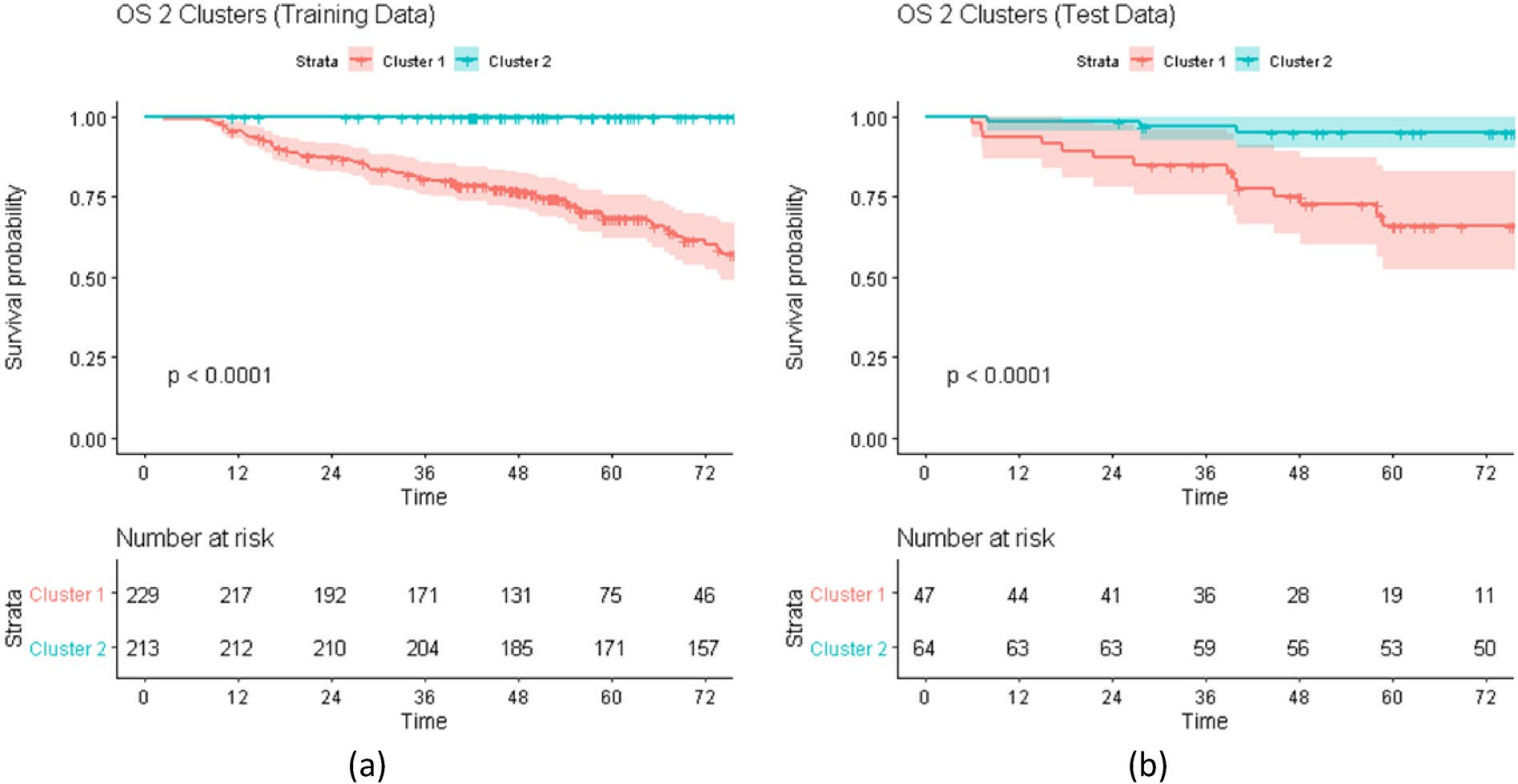
Kaplan–Meier (KM) Curves for Overall Survival (OS). The figure shows the KM curves for OS outcome stratified by the cluster label over (**a**) training and (**b**) test data. For the training, the patients were grouped using Hierarchical Clustering over the co-occurrence matrix from the Random Survival Forest. For the testing, the patients were assigned to a cluster by applying the regression model trained for predicting the cluster labels using the top radiomic features identified by the random survival forest. For both training and testing, the KM curves are significantly different which indicates that the proposed clustering is effective in identifying a risk stratification and can be effectively used as a predictive covariate.

**Figure 3:**
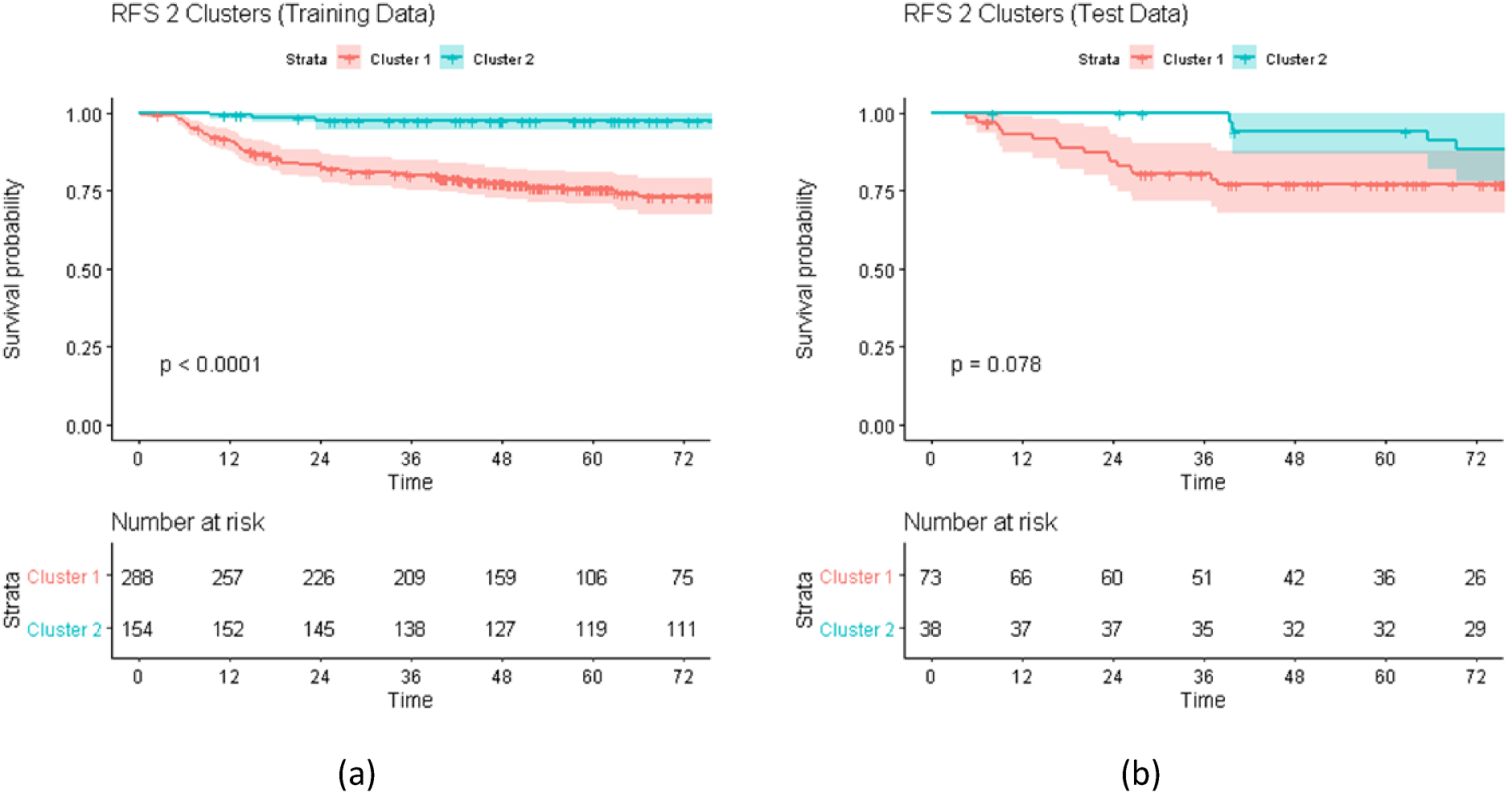
Kaplan–Meier (KM) Curves for Recurrence Free Survival (RFS). The figure shows the KM curves for RFS outcome stratified by the cluster label over (**a**) training and (**b**) test data. For the training, the patients were grouped using Hierarchical Clustering over the co-occurrence matrix from the Random Survival Forest. For the testing, the patients were assigned to a cluster by applying the regression model trained for predicting the cluster labels using the top radiomic features identified by the random survival forest. For both training and testing, the KM curves show two consistent risk groups which indicates that the proposed clustering can be effectively used as a predictive covariate within a risk prediction model.

Figures 2 and 3 shows the Kaplan–Meier survival curves for the training and test patients stratified by the proposed cluster labels for OS and RFS, respectively. These results show that the similarity and the subsequent hierarchical clustering are sensible means to capturing radiomic feature differences. For both clustering outcomes, there is a visible separation between the groups.

Figure 2 shows the curves of the OS outcome for 2 clusters. As can be seen, for both training and testing results, the proposed approach is successful in stratifying the patients by their survival risk. The survival curves for the two clusters are significantly different for both training and testing (*p* value < 0.001).

Figure 3 above shows the curves of the RFS outcome for 2 clusters. As can be seen there is separation between the curves for both the training and testing sets. While the training shows significantly different curves (*p* value < 0.0001), the separation between the survival curves for two clusters over the test dataset is not as statistically significant (*p* value = 0.078).

The supervised feature selection algorithms were used for comparison with the cluster label. The top ranked features using variable importance (highest frequency) for the Random Survival Forest were selected for both OS and RFS. Models including the top 3, 5, and 10 covariates are included.The Cox Proportional Hazards Model using Regularization Paths for Generalized Linear Models via Coordinate Descent (coxnet) identified 5 and 8 radiomic features for OS and RFS, respectively. Table 2 lists all the features names used as predictive covariates in the ensemble model.

**Table 2:** Covariates used in the ensemble model.

Name	Count	Covariates
Clinical	7	Age, HPV status (positive | negative | unknown), Smoking Status (never | former | current), T.category ([T1-T2],[T3-T4]), N.category ([N0-N1],[N2-N3]), Therapeutic Combination (RT alone, Concurrent Chemotherapy (CC), Induction + RT, Induction + CC), AJCC Stage (8th edition)
RSF (OS)	Up to 10	F4.GrayLevelRunLengthMatrix25..90ShortRunLowGrayLevelEmpha,F48.GrayLevelCooccurenceMatrix25180.2ClusterProminence,F48.GrayLevelCooccurenceMatrix25270.1Contrast,F48.GrayLevelCooccurenceMatrix25225.7ClusterShade,F29.IntensityDirectLocalRangeMax,F2.GrayLevelCooccurenceMatrix25270.1Contrast,F2.GrayLevelCooccurenceMatrix25.333.4Correlation,F2.GrayLevelCooccurenceMatrix25180.6MaxProbability,F4.GrayLevelRunLengthMatrix25..90RunLengthNonuniformity,F4.GrayLevelRunLengthMatrix25.333ShortRunEmphasis
RSF (RFS)	Up to 10	F48.GrayLevelCooccurenceMatrix25180.2ClusterProminence,F48.GrayLevelCooccurenceMatrix25315.6ClusterProminence,F8.IntensityDirectKurtosis, F9.IntensityDirectSkewness,F11.IntensityDirectKurtosis, F13.IntensityDirectEnergy,F48.GrayLevelCooccurenceMatrix25180.1InverseDiffNorm,F2.GrayLevelCooccurenceMatrix25180.5ClusterProminence,F2.GrayLevelCooccurenceMatrix25180.5ClusterShade,F14.IntensityDirectEnergy
COX (OS)	5	F25.ShapeVolume, F29.IntensityDirectLocalRangeMax,F4.GrayLevelRunLengthMatrix25..90RunLengthNonuniformity,F6.IntensityDirectSkewness,F48.GrayLevelCooccurenceMatrix25225.7AutoCorrelation
COX (RFS)	8	F5.IntensityDirectGlobalMax, F13.IntensityDirectGlobalMax,F14.IntensityDirectGlobalMax, F25.ShapeVolume,F29.IntensityDirectLocalRangeMax,F4.GrayLevelRunLengthMatrix25..90RunLengthNonuniformity,F4.GrayLevelRunLengthMatrix25..90ShortRunLowGrayLevelEmpha,F48.GrayLevelCooccurenceMatrix25225.7AutoCorrelation
Cluster	1	Cluster label with 2, 3, or 4 values

The clinical covariates are used independently of the outcome being evaluated. Since Random Survival Forests (RSF) and Coxnet (COX) can be used as supervised feature selection methods, the radiomic features selected depend on the outcome used. The top covariates from RSF are selected for each outcome. For COX, the features selected depend on the number of non-zero weights learned by the regularization coefficient. COX selected 5 and 8 radiomics features for OS and RFS, respectively. Cluster refers to the cluster label extracted using Random Survival Forest Clustering.

**Figure 4:**
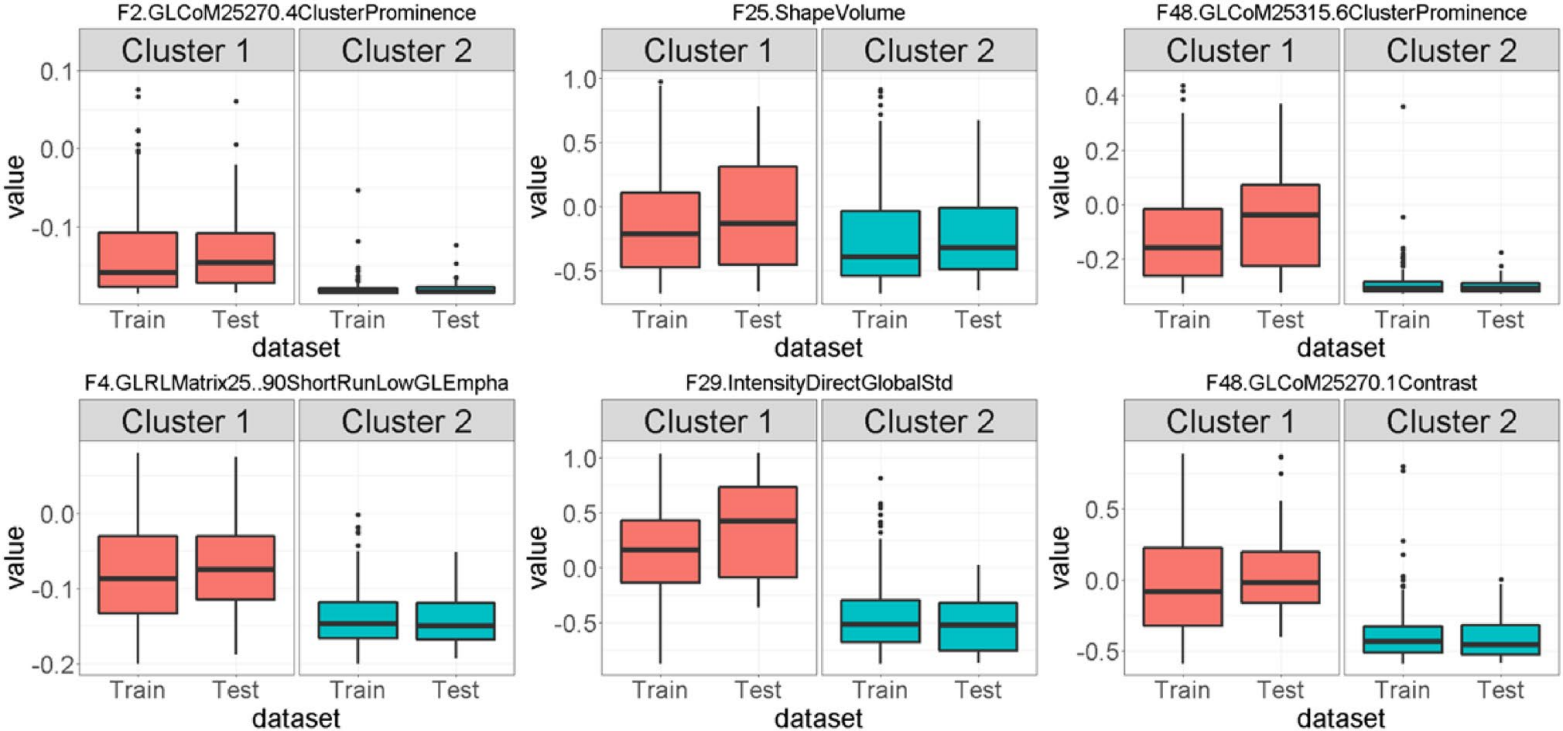
Top Radiomic Features identified by the Random Survival Forest (RSF) for Overall Survival (OS). Boxplots of top 6 features selected using the variable importance from the Random Survival Forest (RSF) over the training data and their distribution within the two clusters identified for Overall Survival (OS). The difference in distribution suggests that these variables can be used in a model to assign cluster labels to test patients. Radiomic features names have been abbreviated to fit in the figure: GL = GrayLevel, CoM = CoocurrenceMatrix, RL = RunLength.

Figure 4 shows the boxplot for the top nine radiomic features for OS within each cluster for training and test data. From the figure it can be seen that the distribution of these features is different between the two clusters, which makes them good candidates for relevant features to train a cluster assignment model to label the test samples. A similar result can be seen in the box plots for RFS outcome (See Appendix A, Fig. A1).

**Figure 5:**
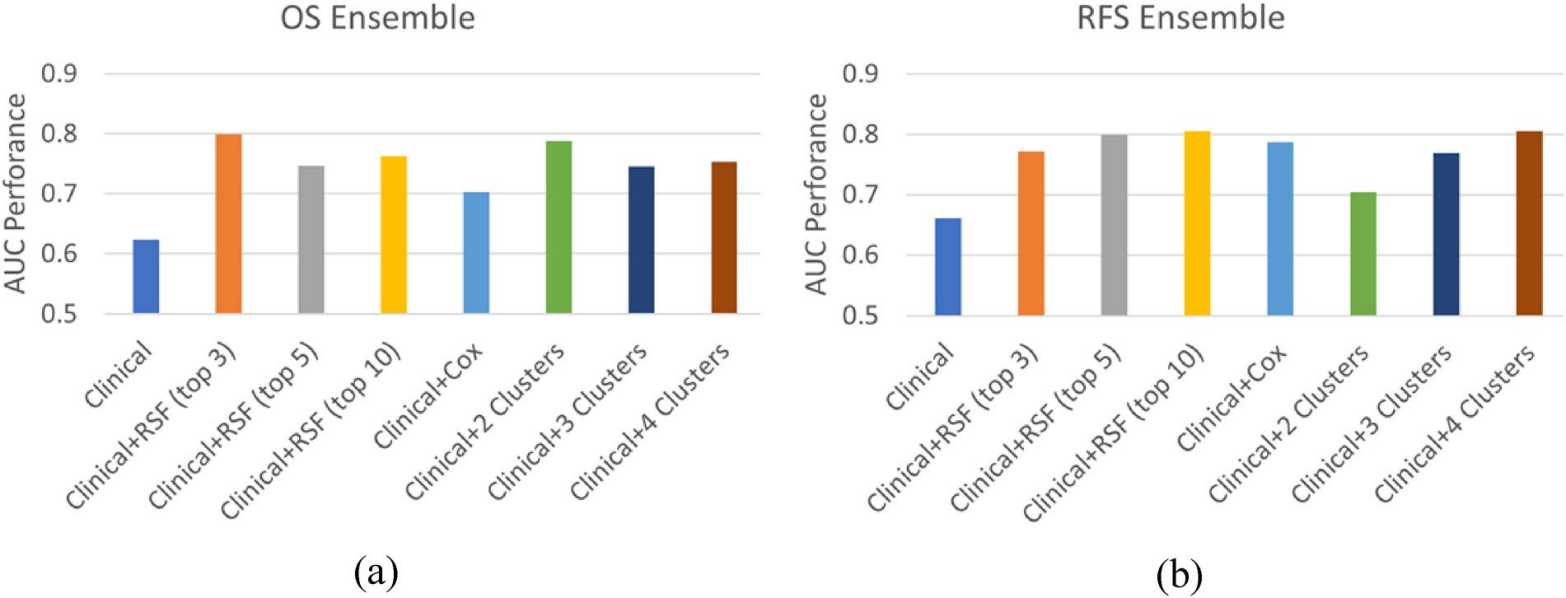
Ensemble model performance over test data. The ensemble model discrimination was evaluated using the AUC metric over the test data for two survival outcomes: (**a**) Overall Survival (OS) and (**b**) Recurrence Free Survival (RFS). Comparison is done between a Clinical baseline model using seven clinical covariates: age, hpv status, smoking status, T-category, N-category, therapeutic combination, AJCC staging, and the models including additional model covariates: selected radiomic features (Clinical + rsf/ + cox), and the proposed cluster labels (Clinical + N Clusters). In all cases, the inclusion of the cluster labels outperforms the Clinical model. The models including the cluster labels show comparable performance to the models including a subset of radiomic features while being considerably more parsimonious models.

Figure 5 compares the ensemble AUC performance for the different predictive covariates over the hold-out test dataset for (a) OS and (b) RFS outcomes. The baseline model is denoted as Clinical and is the one trained using the six clinical covariates: Age, HPV status, Smoking status, Therapeutic Combination, T-Category, and N-Category (8th edition). The Clinical + AJCC.8 model is the baseline model when AJCC Staging (8th edition) is added as a predictive covariate. Clinical + rsf (top n) denotes the baseline model when the top n RSF selected radiomic features are also included in the model. Clinical + cox represents the ensemble where the coxnet selected features have been added to the model. Finally, Clinical + N Clusters is the ensemble model when the radiomic cluster (with N groups) has been added as a predictive covariate. When only clinical covariates are used, AUC over the test data is 0.62 and 0.66 for OS and RFS, respectively. Compared to clinical only, models that incorporated the cluster label (Clinical + 2 Clusters and Clinical + 3 Clusters) as a covariate led to substantial improvement in discrimination. The inclusion of three radiomics derived clusters improves performance by over 17% and 14% (AUC = 0.79 and 0.80) for OS and RFS, respectively. Compared to models which incorporated selected radiomic features directly (+ RSF and + COX), discrimination performance was comparable within 1%.

Table 3 shows the ensemble performance using C-Index and AUC over training and test data for both outcomes. It is worth noting that while Clinical + rsf (top n) and Clinical + coxresults are comparable and close to the Clinical + N Clusters models, the latter is a more parsimonious model.

**Table 3:** Ensemble discrimination performance over training and testing data.

Covariates used in model	Overall survival (OS)				Recurrence free survival (RFS)			
	C-index		AUC		C-index		AUC	
	Train	Test	Train	Test	Train	Test	Train	Test
Clinical	.66	.62	.66	.62	.63	.64	.70	.66
Clinical + rsf (top 3)	.72	**.75**	.75	**.80**	.65	.73	.72	.77
Clinical + rsf (top 5)	.73	.71	.76	.75	.65	.75	.71	.80
Clinical + rsf (top 10)	.76	.71	.80	.76	.66	.75	.72	.80
Clinical + cox	.73	.67	.76	.70	.67	.73	.75	.79
Clinical + 2 Clusters	.81	.75	.85	.79	.73	.66	.82	.70
Clinical + 3 Clusters	.81	.72	.86	.75	.79	.72	.88	.77
Clinical + 4 Clusters	.87	.74	.92	.75	.91	**.75**	.95	**.80**

Comparison of ensemble performance over Train and Test data using C-Index and AUC for both OS and RFS outcomes. Each row in the table corresponds to the ensemble model using different covariates. The Clinical baseline is the model where only clinical covariates are included. The subsequent rows include additional covariates into the baseline model: top n selected radiomic features using rsf (+ rsf (top n)), selected radiomic features using coxnet (+ cox), and the proposed cluster labels (Clinical + N Clusters). The best test results are highlighted in bold. The best test results for OS are obtained by the Clinical + rfs (top 3) (C-Index: .75, AUC: .80) while the best test results for RFS are obtained by Clinical + 4 Clusters (C-Index: .75, AUC: .80).

## Discussion

The proposed method for clustering the high-dimensional radiomic features using hierarchical clustering over the co-occurrence matrix extracted from a Random Survival Forest (RSF) model is a sensible way to summarize the radiomic features into a single covariate. The hierarchical clustering method is robust and generates informative clusters across the different outcomes. The use of a regression model over the most important (frequent) variables selected from the RSF to assign a cluster label offers a simple yet effective way to label the previously unseen test samples. For OS, the Kaplan–Meier survival curves show statistically significant separation between the curves for both training and testing (*p* value < 0.001, Fig. 2). For RFS, even when the test curves follow the same risk stratification as the training curves, the separation between the curves is not as statistically significant (*p* value = 0.078, Fig. 3). A possible explanation for this performance for RFS may be due to the fact that RFS is a combined outcome and only the radiomic features from the primary tumor were considered for clustering. Nevertheless, as can be seen in the model evaluation, the addition of the RFS clusters to other predictive clinical covariates including N-staging, HPV status, and Therapeutic combination improves model performance for both training and testing. Prior work has also effectively leveraged clustering to improve outcome prediction for OPC patients^28–31^, however, none of these works have attempted to use the entire set of radiomic features or Random Survival Forest learning as we have done in this work.

Including the proposed cluster labels as a predictive covariate considerably improves model discrimination for survival outcomes when compared to the same model using clinical data only. Moreover, the performance for models including the radiomic clusters is comparable to the models including radiomic features selected using supervised algorithms (RSF and Coxnet). Several studies on head and neck cancer data have identified radiomic signatures using machine learning approaches to improve different survival outcomes^37,38^. While these algorithms select a small number of radiomic features (up to 10 continuous variables in our experiments), the number of radiomic features is still sometimes larger than the clinical covariates included in the model. In contrast, our cluster label approach yields a more parsimonious model that uses one categorical variable with only 2 or 3 values. Having a smaller subset of features to represent the radiomics is especially useful when there is a small to moderate number of samples, the event rate is low (e.g., under 20% in our case), and few clinical covariates are added into the model (7 in our case).

The results show that a single covariate to represent the high-dimensional radiomic features can be as predictive as a handful of selected radiomic features. Moreover, the cluster label could offer a better generalization by reducing the noise and sparsity of the data. Furthermore, with the proposed method, we are able to easily analyze and stratify the populations based on their cluster labels. An additional benefit of random survival forest clustering is that no feature scaling is required because random forest algorithms are not affected by monotonic transformations. Since the output is a categorical cluster label no scaling is required when training any models either. With feature selection, scaling may be required during selection if methods besides random forest are used either during selection or model training. When a very low-dimensional explanation of radiomic data is required, we recommend the use of dimensionality reduction via random forest clustering, and furthermore, we recommend hierarchical clustering to obtain reasonably balanced clusters.

The main limitations of this work derive from the small sample size and the large number of right-censored samples. Because of these factors, we are not able to evaluate the proposed dimensionality reduction with a large number of clusters or conclude anything about the optimal cluster size. Instead, the number of clusters was varied from 2, because it is the fewest number of clusters, up to 4 because of the categorization of the primary tumor, T category, which typically has 4 categories and because our radiomic feature set is based on the primary tumor. However, while the results were comparable between 2 and 3 clusters, 4 clusters suffered from overfitting in our experiments. Some radiomic clustering studies have used techniques to determine an optimal number of clusters using Principal Component Analysis (PCA) and cluster validation^29^ or consensus clustering^7,8^. In Zdilar et al.^39^, different transformations for right-censored survival outcomes are considered, one of which consists of using the Martingale residuals obtained from a Cox Proportional Hazards model. The Martingale residual can be considered as a continuous outcome. As a potential future work, we could use the Martingale residuals as an outcome and apply the same methodology described in this work using Random Regression Forests^41^ to generate a patient-to-patient similarity matrix for clustering. Another limitation is data availability. Performance status was not collected as part of the dataset and there was a large number of patients with unknown HPV status. While data was not imputed to minimize potential biases, unknown was included into the models as an additional category for HPV status. Furthermore, a large fraction of patients (over 60%) were HPV positive and less than 10% were HPV negative. These factors could limit the predictive power of HPV status.

The use of data from a single institution is another limitation. While the radiomics can be considered homogenous within a single institution, this may not necessarily be the case with data from other institutions where the use of different scanners, voxel sizes, and other factors, may affect the general reproducibility of the proposed model. Therefore, external validation is needed as future work.

In conclusion, dimensionality reduction via random survival forest clustering greatly reduces the radiomic feature space and compares well to feature selection in predictive performance for survival outcomes. This dimensionality reduction can be particularly beneficial when it is desirable to have a very concise representation of the radiomic feature space such as when the number of features is low, or the number of clinical features is already high and the number of samples is moderate to low.

## Supplementary Information

Supplementary Information.

## Data Availability

The datasets analyzed during the current study are available from Scientific Data^42^ and TCGA.
