# Supplementary material for: Oropharyngeal cancer patient stratification using random forest based-learning over high-dimensional radiomic features
Source: Sci Rep. 2021 Jul 7;11:14057. doi: 10.1038/s41598-021-92072-8 (PMC8263609; doi:10.1038/s41598-021-92072-8)
Supplement: Supplementary file 1 — Supplementary Information. [file 41598_2021_92072_MOESM1_ESM.docx]

**APPENDIX A - Supplemental Results**

Figure A1 shows the corresponding boxplot for the top 6 features used for cluster assignment of test patients for Recurrence Free Survival (RFS) outcome.

*Figure A1.* ***Top Radiomic Features identified by the Random Survival Forest (RSF) for Recurrence Free Survival (RFS).*** *Boxplots of top 6 features selected using the variable importance from the Random Survival Forest (RSF) over the training data and their distribution within the two clusters identified for Recurrence Free Survival (RFS). The difference in distribution suggests that these variables can be used in a model to assign cluster labels to test patients. Radiomic features names have been abbreviated to fit in the figure: GL=GrayLevel, CoM=CoocurrenceMatrix, RL=RunLength, Info=Information.*


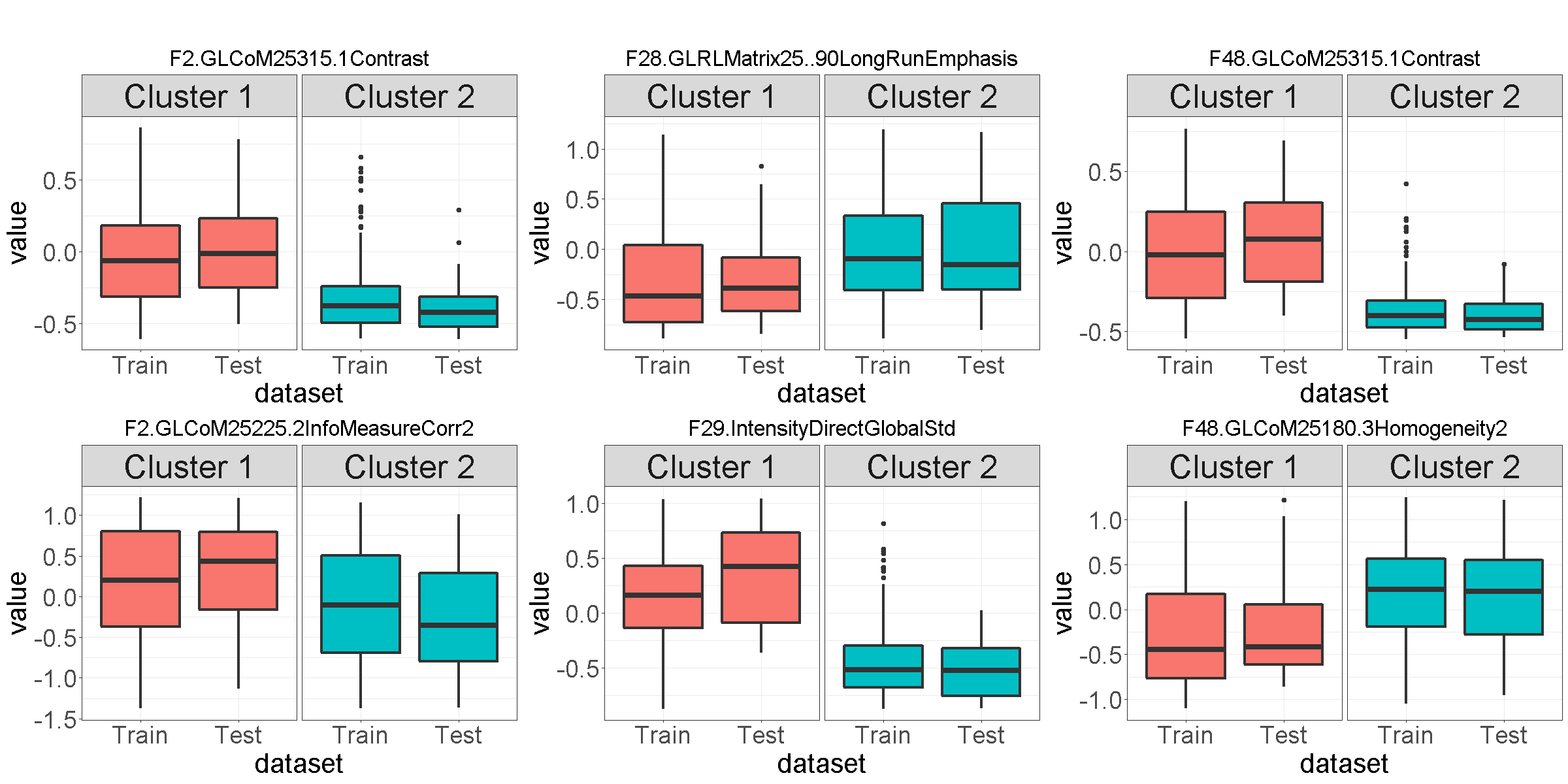


**Appendix B: Cox model results**

As the ensemble model is using 5 different predictive models for outcome prediction it is hard to provide a global model evaluation beyond C-Index and AUC (as included in the paper). In this appendix, we present a more comprehensive analysis for the Cox Proportional Hazard (CoxPH) Model and the effect of including the cluster label as a predictive covariate as CoxPH is so commonly used for survival outcome prediction.

Figures B1 and B2 show the model performance and hazard ratios for the Cox model trained using clinical covariates plus the cluster label for Overall Survival (OS) and Recurrence Free Survival (RFS), respectively. As can be seen, the addition of the cluster label significantly improves model performance.

Figure B3 shows the AUC performance of the ensemble model over the test data when adding the radiomic cluster as a covariate for predicting 5-year survival for (a) OS and (b) RFS. When the cluster labelis included in the model, the AUC performance increase is 28% for OS (2 Clusters) and 22% for RFS (4 Clusters), a substantial improvement in discrimination. The models including the cluster labels, also performed better than the models that incorporated selected radiomic features directly (+RSF (top n) and +COX).

*Figure B1. The Hazard Ratios obtained for OS outcome over the training data when the clinical covariates (age, hpv status, smoking status, T category, N category, Therapeutic Combination, AJCC Stage (8th edition)) and the cluster label are included in a Cox Proportional Hazards Model. As can be seen, even after including the clinical features into the model, the addition of the cluster label is still significant.*


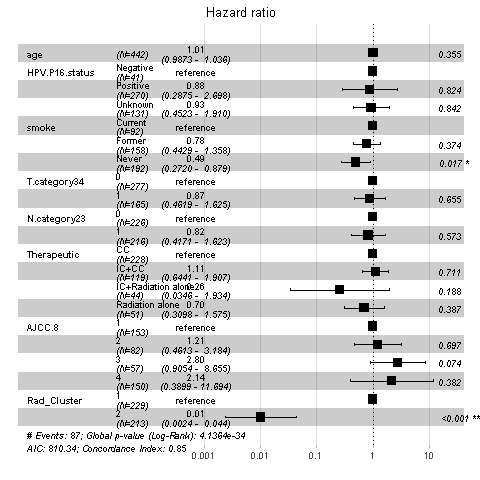


*Figure B2. The Hazard Ratios obtained for RFS outcome over the training data when the clinical covariates (age, hpv status, T category, N category, Therapeutic Combination) and the cluster label are included in a Cox Proportional Hazards Model. As can be seen, even after including the clinical features into the model, the addition of the cluster label is still significant.*


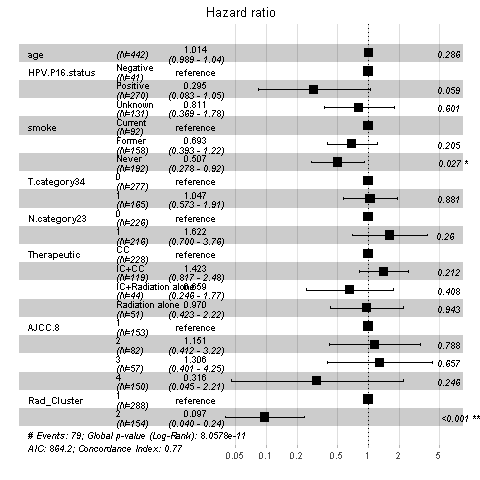


*Figure B3. The AUC evaluation for the cluster labels using the Cox model over the test data for both (a) OS and (b) RFS outcomes when only clinical covariates are included in the model (Clinical) versus includingselected radiomic features (Clinical+RSF (top n)/+COX), and the proposed cluster labels (Clinical+N Clusters).*

| 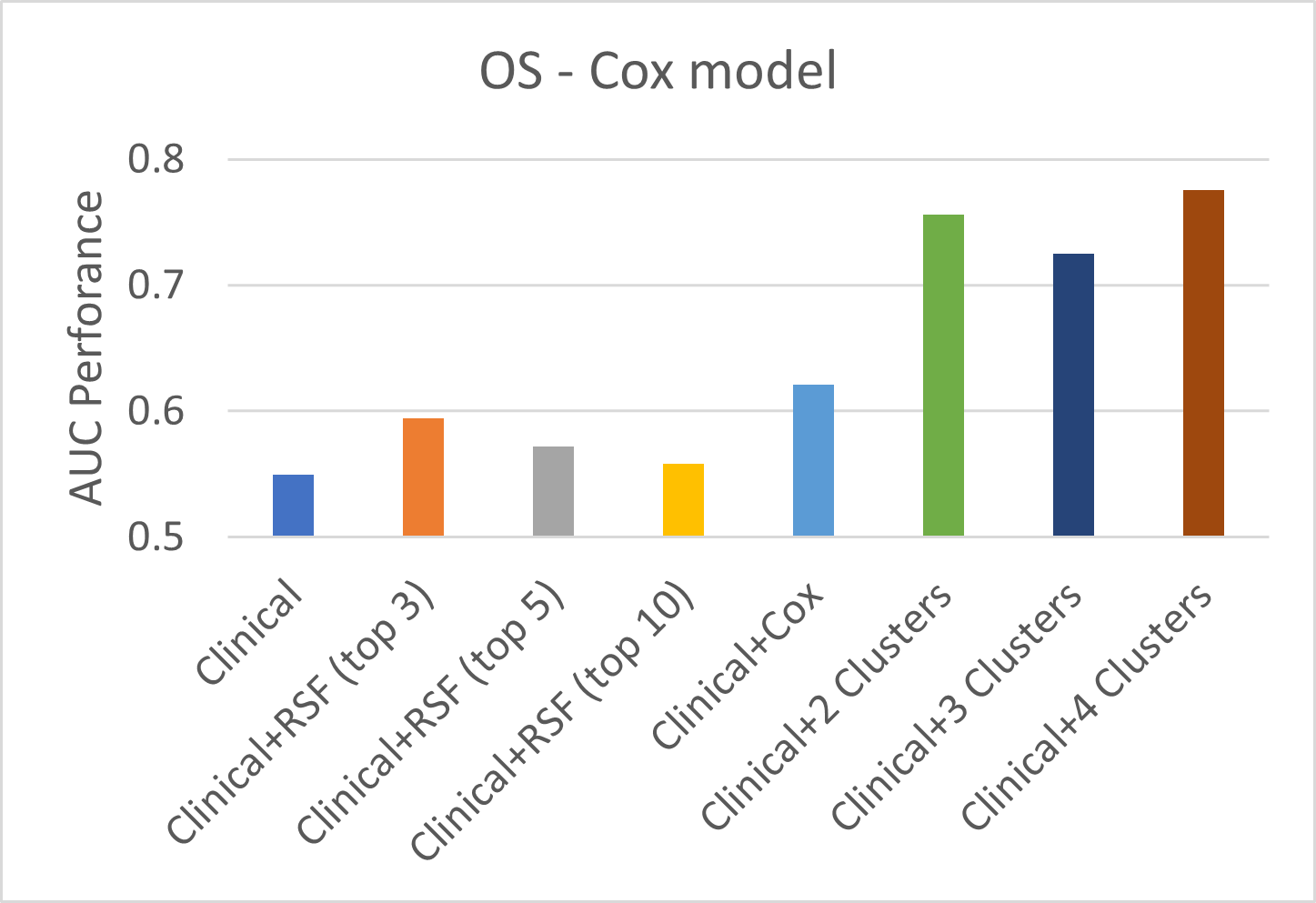(a) | 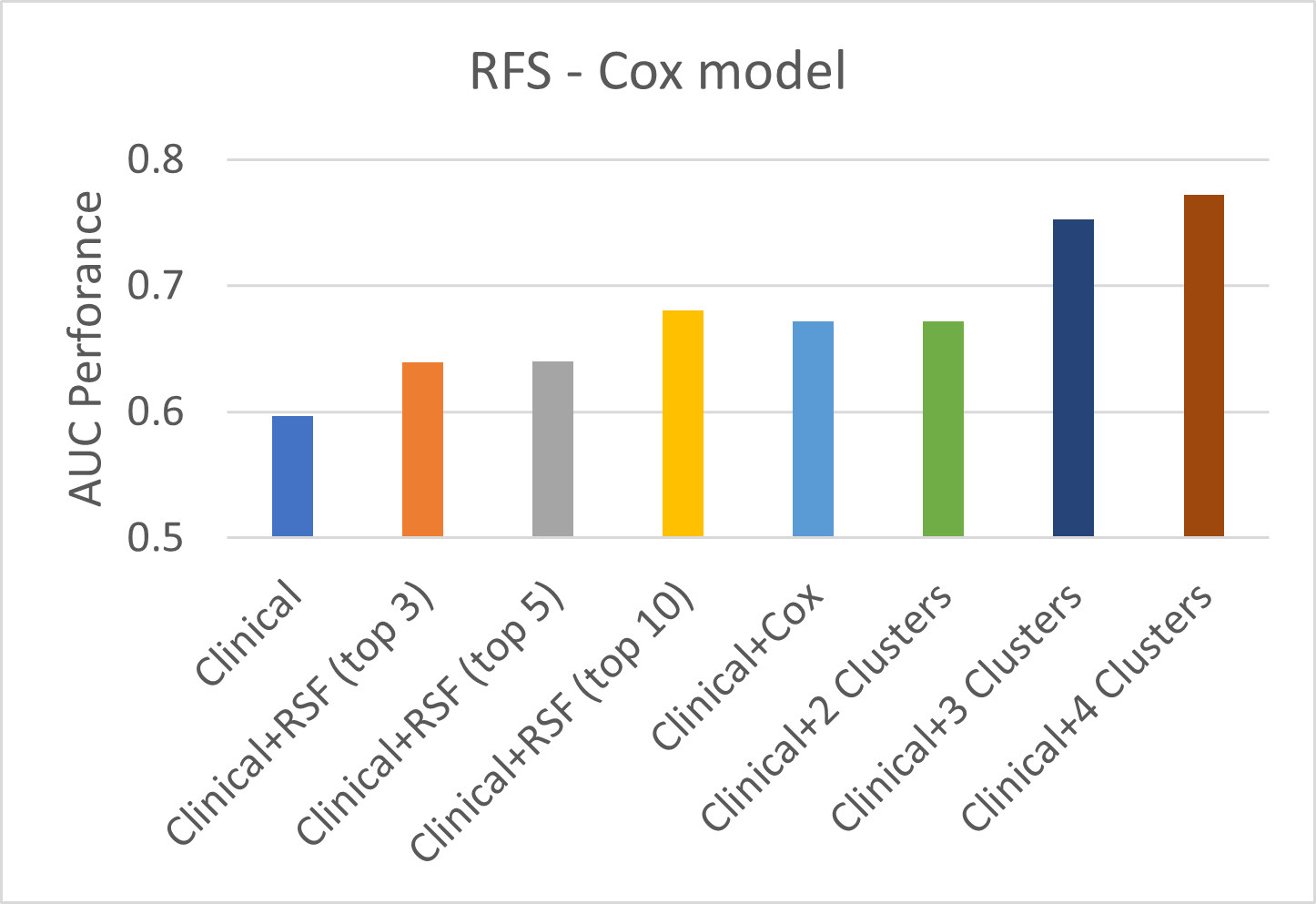(b) |
| --- | --- |

Appendix C: Cox model results

Table C1. Computed tomography- derived intensity histogram, shape and texture analysis features set

| Feature Category | Features | Definition | Ref |
| --- | --- | --- | --- |
| Gray Level Cooccurrence Matrix 25    Gray Level Cooccurrence Matrix 3 | Auto-Correlation | The Correlation texture measures the linear dependency of grey levels on those of neighbouring pixels. | 1 |
|  | Cluster Prominence | A measure of the skewness or asymmetry | 1 |
|  | Cluster Shade | A measure of the skewness or asymmetry | 1 |
|  | Cluster Tendency | Assess if non-random structure exists in the data by measuring the probability that the data is generated by a uniform data distribution | 1 |
|  | Contrast | Returns a measure of the intensity contrast between a pixel and its neighbor over the whole image. | 2, 3 |
|  | Correlation | Returns a measure of how correlated a pixel is to its neighbor over the whole image. | 2, 3 |
|  | Difference Entropy |  | 2, 4 |
|  | Dissimilarity |  | 1 |
|  | Energy |  | 2, 3 |
|  | Entropy |  | 1 |
|  | Homogeneity |  | 2, 3, 4 |
|  | Homogeneity 2 |  | 2, 3, 4 |
|  | Information Measure Correlation 1 |  | 2, 3, 4 |
|  | Information Measure Correlation 2 |  | 2, 3, 4 |
|  | Inverse Diff Moment Norm |  | 2, 3, 4 |
|  | Inverse Diff Norm |  | 2, 3, 4 |
|  | Inverse Variance |  | 4 |
|  | Max Probability |  | 1 |
|  | Sum Average |  | 2, 3, 4 |
|  | Sum Entropy |  | 2, 3, 4 |
|  | Sum Variance |  | 2, 3, 4 |
|  | Variance |  | 4 |
| GrayLevelRunLengthMatrix25 | Gray Level Non-uniformity |  | 5 |
|  | High Gray Level Run Empha |  | 5 |
|  | Long Run Emphasis |  | 5 |
|  | Long Run High Gray Level Empha |  | 5 |
|  | Long Run Low Gray Level Empha |  | 5 |
|  | Low Gray Level Run Empha |  | 5 |
|  | Run Length Non-uniformity |  | 5 |
|  | Run Percentage |  | 5 |
|  | Short Run Emphasis |  | 5 |
|  | Short Run High Gray Level Empha |  | 5 |
|  | Short Run Low Gray Level Empha |  | 5 |
| Neighbor Intensity Difference 25    Neighbor Intensity Difference 3 | Busyness |  | 6 |
|  | Coarseness |  | 6 |
|  | Complexity |  | 6 |
|  | Contrast |  | 6 |
|  | Texture Strength |  | 6 |
| Intensity Direct | Energy |  | 4 |
|  | Global Entropy | The intensity entropy among all the voxels | 4 |
|  | Global Max | The intensity maximum among all the voxels. | 4 |
|  | Global Mean | The intensity mean among all the voxels. | 4 |
|  | Global Median | The intensity median among all the voxels. | 4 |
|  | Global Min | The intensity minimum among all the voxels. | 4 |
|  | Global Std | The intensity standard deviation among all the voxels. | 4 |
|  | Global Uniformity | The intensity uniformity among all the voxels. | 4 |
|  | Inter-Quartile Range | The interquartile range of the intensity values among all the voxels. | 4 |
|  | Kurtosis | Measure the peakedness of all the voxels' intensity. | 4 |
|  | Local Entropy Max | First, at each voxel, compute entropy in its neighborhood region. Then, compute the maximum among all the voxel's entropy calculated from step 1. | 4 |
|  | Local Entropy Mean | First, at each voxel, compute entropy in its neighborhood region. Then, compute the mean among all the voxel's entropy calculated from step 1. | 4 |
|  | Local Entropy Median | First, at each voxel, compute entropy in its neighborhood region. Then, compute the median among all the voxel's entropy calculated from step 1. | 4 |
|  | Local Entropy Min | First, at each voxel, compute entropy in its neighborhood region. Then, compute the minimum among all the voxel's entropy calculated from step 1. | 4 |
|  | Local Entropy Std | First, at each voxel, compute entropy in its neighborhood region. Then, compute the standard deviation among all the voxel's entropy calculated from step 1. | 4 |
|  | Local Range Max | First, at each voxel, compute range value (Max Value-Min Value) in its neighborhood region. Then, compute the median among all the voxel's range value calculated from step 1. | 4 |
|  | Local Range Mean | First, at each voxel, compute range value (Max Value-Min Value) in its neighborhood region. Then, compute the mean among all the voxel's range value calculated from step 1. | 4 |
|  | Local Range Median | First, at each voxel, compute range value (Max Value-Min Value) in its neighborhood region. Then, compute the median among all the voxel's range value calculated from step 1. | 4 |
|  | Local Range Min | First, at each voxel, compute range value (Max Value-Min Value) in its neighborhood region. Then, compute the minimum among all the voxel's range value calculated from step 1. | 4 |
|  | Local Range Std | First, at each voxel, compute range value (Max Value-Min Value) in its neighborhood region. Then, compute the standard deviation among all the voxel's range value calculated from step 1. | 4 |
|  | Local Std Max | First, at each voxel, compute standard deviation in its neighborhood region. Then, compute the maximum among all the voxel's standard deviation value calculated from step 1. | 4 |
|  | Local Std Mean | First, at each voxel, compute standard deviation in its neighborhood region. Then, compute the mean among all the voxel's standard deviation value calculated from step 1. | 4 |
|  | Local Std Median | First, at each voxel, compute standard deviation in its neighborhood region. Then, compute the median among all the voxel's standard deviation value calculated from step 1. | 4 |
|  | Local Std Min | First, at each voxel, compute standard deviation in its neighborhood region. Then, compute the minimum among all the voxel's standard deviation value calculated from step 1. | 4 |
|  | Local Std Std | First, at each voxel, compute standard deviation in its neighborhood region. Then, compute the standard deviation all the voxel's standard deviation value calculated from step 1. | 4 |
|  | Mean Absolute Deviation | The mean absolute deviation of the intensity values among all the voxels. | 4 |
|  | Median Absolute Deviation | The median absolute deviation of the intensity values among all the voxels. | 4 |
|  | Percentile | Percentiles of the intensity values among all the voxels. | 4 |
|  | Quantile | Quantiles of the intensity values among all the voxels. | 4 |
|  | Range | The intensity range (Max Value-Min Value) among all the voxels. | 4 |
|  | Root Mean Square |  | 4 |
|  | Skewness | Measure the asymmetry of all the voxels' intensity. | 4 |
|  | Variance |  | 4 |
| Intensity Histogram | Inter-Quartile Range | The interquartile range of the occurrence probability values in the histogram. | 4 |
|  | Kurtosis | Measure the peakedness of the occurrence probability values in the histogram. | 4 |
|  | Mean Absolute Deviation | The mean absolute deviation of the occurrence probability values in the histogram. | 4 |
|  | Median Absolute Deviation | The median absolute deviation of the occurrence probability values in the histogram. | 4 |
|  | Percentile | Percentiles of the occurrence probability values in the histogram. | 4 |
|  | Percentile Area | Percentiles of values in the accumulative histogram. | 4 |
|  | Quantile | Quantiles of the occurrence probability values in the histogram. | 4 |
|  | Range | Measures the range (Max Value-Min Value) of the occurrence probability values in the histogram. | 4 |
|  | Skewness | Measure the asymmetry of the occurrence probability values in the histogram. | 4 |
| Shape | Compactness 1 | Compactness1= (Volume)/(sqrt(pi)*(SurfaceArea)^(2/3) | 4 |
|  | Compactness 2 | Compactness2= 36*pi*(Volume^2)/((SurfaceArea)^3). | 4 |
|  | Convex | Measure the proportion of the pixels in the convex hull that are also in the region. | 4 |
|  | Convex Hull Volume | The mean volume of the 2D convex hulls that are the convex envelopes of each slice's binary mask. | 4 |
|  | Convex Hull Volume 3D | 3D volume of the convex hull that is the convex envelope of binary mask. | 4 |
|  | Mass |  | 4 |
|  | Max 3D Diameter | Max 3D Diameter= largest pairwise Euclidean distance between voxels on the surface of the tumor volume. | 4 |
|  | Mean Breadth | Denotes integral of mean curvature | 4 |
|  | Number Of Voxel | The number of voxels treating the edge voxels differently. | 4 |
|  | Orientation | Measures the angle between the x-axis and the major axis of the ellipse in 2D. | 4 |
|  | Roundness | Measures how much the binary mask is close to circle in 2D. | 4 |
|  | Spherical Disproportion |  | 4 |
|  | Sphericity |  | 4 |
|  | Surface Area | The surface area of the binary mask. | 7 |
|  | Surface Area Density | Surface Area Density= (surface area of the binary mask)/(volume of the binary mask). | 4, 7 |
|  | Volume | The physical volume treating the edge voxels differently. | 7 |

References

1 Soh, L. K. & Tsatsoulis, C. Texture analysis of SAR sea ice imagery using gray level co-occurrence matrices. IEEE Transactions on Geoscience and Remote Sensing 37, 780-795, doi:10.1109/36.752194 (1999).

2 Haralick, R. M., Shanmugam, K. & Dinstein, I. Textural Features for Image Classification. IEEE Transactions on Systems, Man, and Cybernetics SMC-3, 610-621, doi:10.1109/TSMC.1973.4309314 (1973).

3 Haralick, R. M. & Shapiro, L. G. Computer and Robot Vision. (Addison-Wesley Longman publishing Co., Inc., 1992).

4 Aerts, H. J. W. L. et al. Decoding tumour phenotype by noninvasive imaging using a quantitative radiomics approach. Nature Communications 5, 4006, doi:10.1038/ncomms5006 <http://www.nature.com/articles/ncomms5006#supplementary-information> (2014).

5 Xiaoou, T. Texture information in run-length matrices. IEEE Transactions on Image Processing 7, 1602-1609, doi:10.1109/83.725367 (1998).

6 Amadasun, M. & King, R. Texural Features Corresponding to Texural Properties. IEEE Transactions on Systems, Man and Cybernetics 19, 1264-1274, doi:10.1109/21.44046 (1989).

7 Legland, D., Kiêu, K. & Devaux, M.-F. COMPUTATION OF MINKOWSKI MEASURES ON 2D AND 3D BINARY IMAGES. 2011 26, 10, doi:10.5566/ias.v26.p83-92 (2011).
